# Frequency and outcomes of BRAF alterations identified by liquid biopsy in metastatic, non-colorectal gastrointestinal cancers

**DOI:** 10.1093/oncolo/oyaf044

**Published:** 2025-03-31

**Authors:** Amit Mahipal, Leslie Bucheit, Nicole Zhang, Reagan M Barnett, Michael H Storandt, Sakti Chakrabarti

**Affiliations:** University Hospitals Seidman Cancer Center, Case Western Reserve University, Cleveland, OH, United States; Mayo Clinic, Rochester, MN, United States; Guardant Health Inc, Palo Alto, CA, United States; Guardant Health Inc, Palo Alto, CA, United States; Guardant Health Inc, Palo Alto, CA, United States; Mayo Clinic, Rochester, MN, United States; University Hospitals Seidman Cancer Center, Case Western Reserve University, Cleveland, OH, United States

**Keywords:** cholangiocarcinoma, pancreatic cancer, gastric cancer, BRAF, cell-free DNA

## Abstract

**Background:**

Impact of *BRAF* V600E mutations *(BRAF*^V600E^), a poor prognostic factor in metastatic colorectal cancer, is lacking in non-CRC gastrointestinal (GI) cancers including pancreatic (PDAC), gastric/gastroesophageal (GEA), hepatocellular carcinoma (HCC), and cholangiocarcinoma (CCA). Due to tumor-agnostic approvals for patients with *BRAF*^V600E^, understanding the frequency and impact of *BRAF* alterations across non-CRC GI cancers is essential for clinical decision-making.

**Methods:**

Patients with PDAC, GEA, HCC, or CCA who had cell-free DNA detected on Guardant360 (Guardant Health) from 2020 to 2023 were queried. Prevalence of characterized *BRAF* genomic alterations (GA) was calculated; GAs were grouped by class (Class I/II/III). The Chi-squared test assessed differences between cancer types. A subset of patients had outcomes analysis using GuardantINFORM, a real-world clinicogenomic database, to derive real-world overall survival (rwOS).

**Results:**

Of 32 480 included patients, *BRAF* GAs were identified in 4.4%; 19% were *BRAF*^V600E^ (0.81% prevalence overall). CCA had the highest rate of *BRAF* GAs and *BRAF*^V600E^ (*P* < .01); HCC and GEA had the highest rates of *BRAF* class II/III alterations. There were no significant differences in rwOS by alteration class or cancer type; numeric differences were observed by alteration class. Few patients were treated with BRAF inhibitors (2.2%). Prevalence of co-occurring alterations was unique by cancer type.

**Conclusions:**

Frequency of *BRAF* GAs, including *BRAF*^V600E^, in non-CRC GI cancers detected by liquid biopsy is similar to tissue-based rates and can be reliably used to assess *BRAF* status. *BRAF* GAs have mixed prognostic implications on survival for patients with non-CRC GI malignancies that warrant further exploration.

**Implications for practice**

Novel drug therapeutics targeting BRAF alterations are under development. However, the frequency and types of BRAF alteration in non-colorectal gastrointestinal cancers are not well defined. In this study, using liquid biopsy, we found that the highest frequency of class 1 BRAF alterations is present in cholangiocarcinoma while Class II/III BRAF alterations are more common in hepatocellular cancer and gastroesophageal cancer. We did not observe the detrimental effect of BRAF mutations on overall survival in these tumor types. Despite the tumor-agnostic FDA approval of BRAF inhibitors, their utilization was low in clinical practice in non-colorectal gastrointestinal cancers.

## Background

BRAF is a protein kinase that functions in cell signaling pathways impacting cellular growth and differentiation and *BRAF* mutations have been implicated in oncogenic pathways.^1^*BRAF* mutations have been found to have a prognostic impact and may guide therapeutic decisions in patients with multiple solid malignancies, including colorectal cancer (CRC),^2-4^ melanoma,^5,6^ and non-small cell lung cancer (NSCLC).^7^*BRAF*^V600E^ is the most frequently observed and best-studied mutation, however, new studies have evaluated *BRAF*^non-V600E^ mutations as well, identifying distinct phenotypic and prognostic implications of these less frequently observed variants.^8-11^

*BRAF* mutations may be characterized as class I, II, or III. Class I mutations include *BRAF*^V600E^ and result in constitutive activation of BRAF monomers independent of RAS, while class II mutations lead to constitutive activation of BRAF dimers independent of RAS.^8^ Alternatively, class III mutations lead to more avid binding of RAS and CRAF. In patients with CRC, class I mutations (*BRAF*^V600E^) are associated with proximal malignancies that are poorly differentiated, conferring poorer survival.^2,12,13^ More recent studies have sought to better understand *BRAF*^non-V600E^ mutations, which may be observed in approximately 2.2% of CRC cases.^9^ In CRC, class II mutations generally confer a poorer prognosis than *BRAF*^WT^, while class III mutations have similar survival compared to *BRAF*^WT^.^8^ However, *BRAF*^V600E^ and other classes of *BRAF* alterations and their clinical implications have not been broadly assessed in other gastrointestinal (GI) malignancies, including pancreatic cancer (PDAC), gastric/gastroesophageal cancer (GEA), hepatocellular carcinoma (HCC), and cholangiocarcinoma (CCA).^14^

Recently, the United States Food and Drug Administration (FDA) approved the combination of dabrafenib, a BRAF inhibitor, and trametinib, a MEK inhibitor, for advanced solid malignancies harboring *BRAF*^V600E^ mutation. This is based on the results of the ROAR trial, an open-label phase II basket trial that enrolled patients with rare malignancies harboring *BRAF*^V600E^ mutation. Interval analysis demonstrated impressive results in multiple tumor types with overall response rates of 47% in biliary tract cancer,^15^ 56% in anaplastic thyroid cancer,^16^ 33% in high-grade glioma, and 69% in low-grade glioma.^17^ Additionally, recent studies in metastatic CRC demonstrated prolonged preservation in quality of life in those treated with BRAF inhibitors.^18^ As such, this further highlights the importance of better assessing *BRAF*^V600E^ prevalence in expanded cancer types, including non-CRC GI malignancies.

To detect *BRAF* mutations, cell-free DNA (cfDNA) may be used clinically and/or in research studies. Studies have demonstrated high concordance of genomic alterations detected via cfDNA analysis with tissue biopsy in multiple GI malignancies including CRC,^19^ PDAC,^20^ and GEA.^21^ The advantage of cfDNA includes its minimally invasive nature, faster turnaround time, and low failure rate for next-generation sequencing testing. Assessing *BRAF* status via cfDNA may have utility in identifying patients who may have matched therapy or clinical trial options. As such, we sought to evaluate the prevalence of *BRAF*^V600E^ and BRAF^non-V600E^ alterations in non-CRC GI malignancies using cfDNA and report select patient outcomes.

## Methods

### Prevalence assessment

Patients with advanced PDAC, GEA, HCC, or CCA who had cfDNA detected as part of routine clinical testing (Guardant360, Guardant Health) in the United States from October 1, 2020 to September 30, 2023 were retrospectively assessed to determine the prevalence of *BRAF* genomic alteration (GA). Detection of cfDNA may be observed in 85%–90% of patients assessed, depending on tumor shed.^22,23^ Only the first test for patients was included if multiple tests were performed. Guardant360 is a CLIA-certified, CAP-accredited, cfDNA next-generation sequencing (NGS) assay with prior analytical and clinical validations reported.^23,24^ Guardant360 can detect genomic alterations in up to 83 genes and, in select genes, identify copy number amplifications, rearrangement/fusions, and insertions/deletions.

Patients with cfDNA detected were sorted by cancer type and stratified by *BRAF* status: no *BRAF* alteration detected, *BRAF*^V600E^ (class I), *BRAF* class II, *BRAF* class III, or other predicted *BRAF* driver alteration as previously published (listed in Supplementary Table S1).^14^*BRAF* amplifications and/or characterized alterations excluding variants of uncertain significance and synonymous alterations were removed for analysis by class. If patients had more than one *BRAF* alteration, then they were assigned the class with the highest alteration (eg, *BRAF*^V600E^ and *BRAF* class II alterations detected in the same patient would assign the patient to the V600E/class I group). Clinical factors, such as diagnosis, clinical status (newly diagnosed, not responding to therapy), age, and sex were derived from clinician-completed test requisition forms; comparisons across cancer types were assessed using the chi-squared test with *P*-value for the significance of *P* < .05.

### Outcomes analysis: overall survival by braf status

Outcomes of patients with *BRAF* alterations in the prevalence analysis were assessed using GuardantINFORM, a real-world evidence database that includes anonymized genomic data and structured payer claims from inpatient and outpatient settings across academic and community practices. GuardantINFORM does not include clinical features that are not coded as claims (eg, biomarkers assessed by other tests, clinical response to cancer treatment, etc.).

Patient treatments were recapitulated over the study period using claims data in GuardantINFORM. Patients were required to have 2 metastatic diagnosis codes for their cancer to confirm their cancer type and metastatic state as well as capture baseline characteristics. Patients were stratified by cancer type and classified *BRAF* alteration group as performed in the Prevalence cohort for V600E/class I, class II, and class III. Co-occurring alterations were assessed for patients with *BRAF* V600E alterations by cancer type regardless of availability of evaluable outcomes, where genes were altered in at least 1% of the cancer-type specific population. Then, outcomes were assessed for all *BRAF* classes, where patients were required to have treatment and evaluable outcomes (eg, no conflicting death dates). Real-world overall survival (rwOS) was evaluated in months and defined as the time from G360 testing report date until death from any cause; patients were censored at the last claim activity. Log-rank tests were used to analyze differences. Demographics and Elixhauser comorbidity scores were compared across *BRAF* groups for each cancer type using the chi-squared test. Significance was defined as *P* < .05 for all tests. Of note, HCC and “other putative driver *BRAF* alterations had a low number of outcomes available and thus were not included in outcomes analysis.

### Ethics statement

De-identified research datasets generated by Guardant Health are approved by the Adverra IRB with a waiver of consent. The GuardantINFORM database is a fully deidentified database that complies with sections 164.514(a)-(n)1ii of the U.S. Health Insurance Portability and Accountability Act (HIPAA) regarding the determination and documentation of statistically deidentified data.

## Results

### Prevalence and features of BRAF genomic alterations

The study cohort consisted of 32 480 patients with advanced PDAC, GEA, HCC, or CCA who underwent cfDNA testing and had at least one alteration detected in circulating tumor DNA (ctDNA). Median age of the study cohort was 68 years (range: 18-102 years) with male preponderance (58.7%). Clinical status was reported for 89% (*m* = 29,124) with ~65% having cfDNA testing at the time of diagnosis and the remainder (~35%) performed when not responding to therapy (as reported by ordering clinicians); these rates did not differ by tumor type (Table 1).

**Table 1.** Demographics and BRAF prevalence assessment by cancer type.

	Total *n*	PDAC	GEA	CCA	HCC
Cohorts (*n*, %)	32 480	15 578 (48.0)	8435 (26.0)	6189 (19.1)	2278 (7.0)
Male sex (*n*, %)	19 084 (58.7)	8251 (53.0)	6080 (72.1)	3055 (49.4)	1697 (74.5)
Median age, years	68 (18-102)	69 (20-102)	66 (18-100)	68 (18-98)	67 (16-97)
Percent newly diagnosed	64.93	64.18	63.86	66.52	69.54
BRAF alterations (*n*,%)	969 (2.98)	361 (2.3)	219 (2.6)	328 (5.3)	61 (2.7)
*BRAF* V600E (class I)	264 (0.81)	79 (0.51)	53 (0.63)	114 (1.8)	18 (0.79)
*BRAF* class II	256 (0.79)	117 (0.75)	57 (0.68)	69 (1.1)	13 (0.57)
*BRAF* class III	329 (1.01)	103 (0.66)	87 (1.03)	119 (1.9)	20 (0.88)
Other predicted driver	120 (0.37)	62 (0.40)	22 (0.26)	26 (0.40)	10 (0.44)

**Figure 1.**
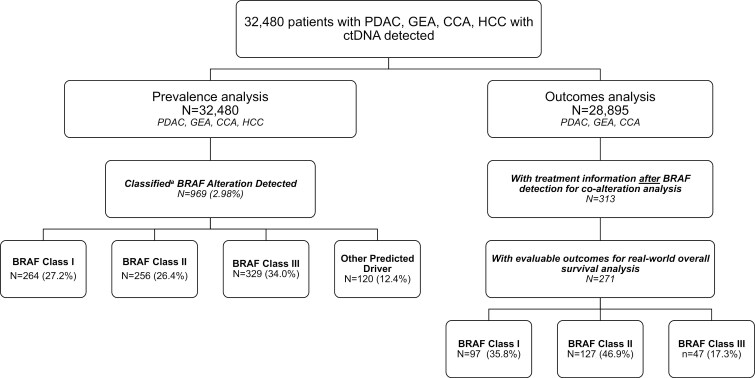
Consort diagram. ^a^Owsley et al.^14^ Abbreviations: CCA, cholangiocarcinoma; GEA, gastroesophageal cancer; HCC, hepatocellular carcinoma, PDAC, pancreatic cancer.

*BRAF* alterations were identified in 1375 (4.4%) patients, of which 407 were amplifications. Of patients who had *BRAF* mutations (*n* = 969), 264 were *BRAF*^V600E^/Class I for an overall prevalence of 0.81% (Figure 1). Twenty-four patients with *BRAF*^V600E^/class I alterations also harbored *BRAF* amplifications (1.7%); 23 patients had MSI-H detected (1.6%). When assessing differences by tumor type, cholangiocarcinoma had the highest rate of *BRAF* alterations (> 5%) including the highest rate of *BRAF*^V600E^/class I alterations compared to other tumor types (Table 1, *P* < .01). Gastroesophageal cancers and HCC had higher rates of class II/III alterations than V600E/class I alterations.

When compared to published rates of classified *BRAF* alterations identified on tissue-based testing,^14^ rates were higher for each cancer type assessed via ctDNA when comparable: 3.8% tissue-based CCA *BRAF* alteration rate compared to 5.3% on ctDNA, 1.12% tissue-based rate for GEA compared to 2.6% on ctDNA, and 1.49% tissue-based rate for PDAC compared to 1.49% for ctDNA. Notably, there was not a tissue-based reference for hepatocellular carcinoma available in the literature, likely due to its diagnostic protocol that may not require tissue biopsy.

## Outcomes analysis

Three-hundred fourteen patients with *BRAF* alterations were assessed using GuardantINFORM for co-alteration analysis (*n* = 155 with CCA, *n* = 67 with GEA, *n* = 92 with PDAC; Supplement Figure S1A-C).

Outcome data were available for 271 patients with class I, II, or III *BRAF* alterations comprising 31.9% of the prevalence cohort (*n* = 100 with CCA, *n* = 95 with PDAC, and *n* = 76 with GEA; Table 2). When assessing *BRAF* alterations by class, higher rates of class II alterations and lower rates of class III alterations were observed in the outcomes cohort compared to the overall prevalence cohort (*P* < .0001).

**Table 2.** Real-world overall survival by cancer type and by BRAF mutational classification.

Cholangiocarcinoma			
	Class I	Class II	Class III
Subjects	46	36	13
Events	19	15	5
Censored	27	21	8
Median rwOS in months	12.47	11.67	10.63
95% CI	10.63-24.57	9.57-NR	9.1-NR
Log-rank *P* across groups	*P* = .45		
Pancreatic cancer			
	Class I	Class II	Class III
Subjects	33	47	20
Events	9	20	**6**
Censored	24	27	14
Median rwOS in months	NR	11.30	NR
95% CI	10.17-NR	7.33-NR	6.10-NR
Log-rank p across groups	*P* = 0.36		
Gastro/esophageal cancer			
	Class I	Class II	Class III
Subjects	18	44	14
Events	4	14	4
Censored	14	29	10
Median rwOS in months	14.70	NR	31.60
95% CI	10.90-NR	8.93-NR	22.67-NR
Log-rank *P* across groups	* P* = .76		

Abbreviations: CI, confidence interval; rwOS: real-world overall survival.

There were no statistically significant differences in real-world overall survival (rwOS) for patients with *BRAF*^V600E^ compared to those without for any cancer type (*P* = 0.17-0.97). When assessing alterations by cancer type, patients with *BRAF*^V600E^ alterations had numerically improved rwOS for CCA and PDAC; the inverse was observed for GEA [CCA *BRAF*^V600E^ median survival: 34.1 months (95% CI, 22.0-46-2) vs 24.8 months (95% CI, 23.9-25.9); PDAC *BRAF*^V600E^ median survival 70.6 months (95% CI, 12.9-NR) vs 20.9 (95%CI, 20.4-21.7); gastroesophageal *BRAF*^V600E^ medial survival: 27.0 (95%CI, 17.8-55.9) vs 29.5 (95% CI, 28.23-31.40). While patients with CCA and *BRAF* Class I (V600E) alterations were significantly younger than those with Class II or III mutations (*P* = .0006, Table 3), there were no other demographic differences within cancer groups across age, sex, comorbidity index, or smoking status.

**Table 3.** Demographics by BRAF mutational class for patients assessed for real-world overall survival

Cholangiocarcinoma				
	BRAF class I (V600E, *n* = 46)	BRAF class II (*n* = 36)	BRAF class III (*n* = 13)	*P-*value
Age in years				0.0006
Median	58.5	67.5	69	
<65 (*n*, %)	33 (71.7)	14 (38.9)	3 (23.1)	
65-74 (*n*, %)	7 (17.4)	11 (30.6)	7 (53.9)	
75+ (*n*, %)	5 (10.9)	11 (30.6)	3 (23.1)	
Sex				0.85
Male, *n* (%)	20 (43.5)	17 (47.2%)	5 (38.5)	
Comorbidity				
ECI, mean (SD)	5.3 (2.5)	4.9 (3.2)	5.8 (3.4)	0.38
ECI, median	3	3	3	
Smoking				
Ever smoker, *n* (%)	10 (21.7)	8 (22.2)	4 (30.8)	0.78
Pancreatic cancer				
	BRAF class I (V600E, *n* = 33)	BRAF class II (*n* = 47)	BRAF class III (*n* = 20)	*P*-value
Age				0.72
Median	70	67	66.5	
<65 (*n*, %)	11 (33.3)	18 (38.3)	8 (40.0)	
65-74 (*n*, %)	11 (33.3)	13 (27.8)	10 (50.0)	
75+ (*n*, %)	11 (33.3)	16 (34.0)	2 (10.0)	
Sex				0.08
Male, *n* (%)	15 (45.5)	32 (68.1)	14 (70.0)	
Comorbidity				
ECI, mean (SD)	5.7 (3.0)	5.0 (2.8)	4.9 (1.8)	0.63
ECI, median	5	4	5	
Smoking				
Ever smoker, *n* (%)	6 (18.2)	13 (27.7)	4 (20.0)	0.057
Gastro/esophageal cancer				
	BRAF class I (V600E, *n* = 18)	BRAF class II (*n* = 44)	BRAF class III (*n* = 14)	*P*-value
Age				0.9
Median	66.6	64.2	64	
<65	8 (44.4%)	18 (40.9%)	8 (57.1%)	
65-74	3 (16.7%)	17 (38.6%)	2 (14.3%)	
75+	7 (38.9%)	9 (20.5%)	4 (28.6%)	
Sex				0.46
Male, *n* (%)	11 (61.1%)	30 (68.2%)	7 (50.0%)	
Comorbidity				
ECI, mean (SD)	5.4 (2.6)	4.9 (3.2)	5.8 (3.4)	0.38
ECI, median	5	4	6	
Smoking				
Ever smoker, *n* (%)	6 (33.3%)	17 (38.6%)	6 (42.9%)	0.86

Abbreviation: ECI, Elixhauser comorbidity index.

Real-world overall survival (rwOS) differed by *BRAF* alteration class across tumor types (Table 2), however no statistically significant differences were noted. For patients with CCA, those with class III alterations had the numerically greatest median rwOS (24.33 months, 95%CI, 9.1-NR) compared to V600E/class I and class II alterations [12.47 months (95% CI, 10.63-24.57); 11.67 months (95%CI, 9.567-NR), respectively]. Notably, only 3/46 (6.5%) patients with V600E/class I alterations were treated with *BRAF* inhibitors; most patients had chemotherapy and/or immunotherapy-containing regimens (Table 4) with the highest rates observed in patients with BRAF class III alterations (78.6%) compared to those with V600E/class I and class II (52.0%, 52.8%, respectively; Table 4).

**Table 4.** Treatments by cancer type and BRAF mutational class in the outcomes cohort (*n*, %)

Cholangiocarcinoma				
Therapy	BRAF class 1 (*n* = 46)	BRAF class 2 (*n* = 36)	BRAF class 3 (*n* = 14)	Total (*n* = 95)
BRAF inhibitor	3 (6.5)	0 (0)	0 (0)	3 (3.2)
Chemo only	24 (52.0)	19 (52.8)	11 (78.6)	54 (56.8)
Chemo + Bevacizumab	1 (2.1)	2 (5.6)	0 (0)	3 (3.2)
Chemo/IO	6 (13.0)	11 (3.1)	1 (7.7)	18 (18.9)
IO only	9 (19.6)	2 (5.6)	1 (7.7)	12 (12.6)
Targeted therapy	3 (6.5)	2 (5.6)	0 (0)	5 (5.3)
Pancreatic cancer				
Therapy	BRAF class 1 (*n* = 33)	BRAF class 2 (*n* = 47)	BRAF class 3 (*n* = 20)	Total (*n* = 100)
BRAF inhibitor	1 (3.0)	0 (0)	0 (0)	1 (1.0)
Chemo only	22 (66.7)	41 (87.2)	20 (100)	83 (83.0)
IO only	8 (24.2)	4 (8.5)	0 (0)	12 (12.0)
Targeted therapy	2 (6.1)	2 (4.2)	0 (0)	4 (4,0)
Gastroesophageal cancer				
Therapy	BRAF class 1 (*n* = 18)	BRAF class 2 (*n* = 44)	BRAF class 3 (*n* = 14)	Total (*n* = 76)
BRAF inhibitor	2 (11.1)	0 (0)	0 (0)	2 (2.6)
Chemo only	8 (44.4)	27 (61.4)	9 (64)	44 (57.9)
Chemo + Bevacizumab	1 (5.6)	1 (2.3)	0 (0)	2 (2.6)
Chemo + anti-HER2	0 (0)	1 (2.3)	1 (7.1)	2 (2.6)
Chemo/IO	4 (22.2)	7 (15.9)	2 (14.2)	13 (17.1)
IO only	2 (11.1)	4 (9.0)	1 (7.1)	7 (9.2)
Targeted therapy	1 (5.6)	4 (9.0)	1 (7.1)	6 (7.9)

For PDAC, median rwOS was numerically poorest for patients with class II alterations (11.30 months, 95% CI, 7.33-NR); however, many patients were censored in other groups (class I, class III). Only one patient of 33 with *BRAF*^V600E^/class I alterations received a *BRAF* inhibitor with >80% of the cohort receiving chemotherapy, as expected. All patients with Class III had chemo-only regimens compared to V600E/class I and class II who had lower rates of chemo-only regimens (Table 4).

Finally, patients with GEA who harbored *BRAF*^V600E^/class I alterations had the numerically poorest median rwOS (14.70 months, 95% CI, 10.90-NR), while those with class III alterations had the numerically highest rwOS (31.60 months, 95% CI, 22.67-NR). Of 18 patients with V600E/class I alterations, 2 (11%) were treated with BRAF inhibitors, the highest rate among cancer types assessed. Patients with V600E/class I alterations had lower rates of chemotherapy-only regimens (44%) and higher rates of chemo/IO (22%) regimens compared to class II and III (chemo-only: 44% vs. 61% vs. 64%, respectively; chemo/IO: 22% vs. 15% vs. 14%, respectively; Table 4).

When assessing co-occurring alterations in patients with *BRAF*^V600E^ (regardless of outcomes availability), certain pathways were observed to be impacted most frequently, including the RTK pathway, p53 cell cycle pathway, and the Wnt/β-catenin pathway (Supplementary Figure S1A-C). This includes frequently harbored alterations in *TP53* (41%), *FGFR2* (12%), *PIK3CA* (12%), and *KRAS* (10%) in *BRAF*^V600E^ CCA (Supplementary Figure S1A); *TP53* (47%), *KRAS* (21%), *APC* (13%), and *SMAD4* (12%) in *BRAF*^V600E^ PDAC (Supplementary Figure S1B); and *TP53* (61%), *APC* (19%), *ARID1A* (19%), and *EGFR* (15%) in *BRAF*^V600E^ GEA (Supplementary Figure S1C). Alterations across these pathways can impact processes such as cell proliferation, cell survival, and differentiation, all contributing to the carcinogenic process.^25,26^

## Discussion

In the present study, we demonstrate the feasibility of cfDNA detection to reliably identify *BRAF* mutations in non-CRC GI malignancies, which may have implications for clinical management. In our study, *BRAF* alterations were identified in approximately 4% of these non-CRC GI malignancies. The frequency of *BRAF* alterations in this group is lower when compared to other malignancies, including CRC (10%)^3,4^ and melanoma (50%).^27^ To our knowledge, this is one of few studies reporting prognostic implications of *BRAF* mutations in non-CRC GI malignancies.

Among GI malignancies evaluated, classified *BRAF* alterations were most frequently observed in CCA, which also had the highest rate of V600E/class I alterations. While differences in rwOS among *BRAF* mutation classes were not statistically significant, a numerical trend towards superior rwOS was noted among those with class III mutations, which is consistent with previously observed positive prognostic value of class III mutations in colorectal cancer; notably low numbers of patients with this type of mutation in CCA does limit analysis to some extent.^28^ Outcomes of those with class I and class II mutations were similar, which is not unexpected considering observations of similar behavior between these 2 mutational classes in patients with CRC.^8^ Other studies evaluating the prognostic implications of *BRAF* alterations in CCA are limited, and this requires further evaluation.

*BRAF* mutations were found in 2.3% of patients with PDAC, which is consistent with prior reports noting an incidence of 2-4% in those with advanced disease.^29^ In the present study, those with class II mutations appeared to have the shortest rwOS, although conclusions are challenging due to the high rates of censorship in this cohort. Notably, of 33 patients with a BRAF^V600E^ mutation, only one received *BRAF*-targeted therapy. Considering the poor prognosis with PDAC and the lack of therapeutic options, this does demonstrate an unmet need.

Lastly, *BRAF* mutations were seen in 2.6% of patients with GEA. Previous studies of gastric or esophageal cancers report a low frequency of 0-1%.^30,31^ A higher frequency of *BRAF* alterations may be observed here due to differences in clinical status compared to other studies (newly diagnosed vs. not responding to therapy) and/or heterogeneity in GEA which has been well documented. While not significant, there was a numerical trend toward longer rwOS in those with class II/III alterations, reflecting the prognostic pattern of CRC, although low patient count does limit this analysis. Baseline characteristics among those with various *BRAF* statuses were not explanatory of the observed differences. One prior study reported *BRAF* overexpression to be correlated with poorer survival in esophageal cancer, however, it is difficult to draw comparisons between this study and the present one.^32^

In addition to defining the prevalence and prognostic implications of *BRAF* mutations in non-CRC GI malignancies, this study demonstrates the ability of ctDNA to reliably characterize somatic *BRAF* mutations. Among patients with cfDNA detected, rates of *BRAF* mutation detection were comparable to those observed via tissue-based assays, suggesting the validity of this liquid biopsy assay.^33,34^ Rates in this study may have been slightly elevated given tissue-based assays are often deployed at the time of diagnosis; this cohort overall was assessed using liquid biopsy after diagnosis in 35% of cases, suggesting perhaps higher rates of *BRAF* due to heterogeneity and/or treatment pressure not captured at tissue biopsy at the time of diagnosis. However, the validity of using liquid biopsy for *BRAF* detection has been supported by additional studies.^35^

Notably, ~1% of the cohort had V600E/class I alterations yet only 6 patients were treated with *BRAF* inhibitors While it is unclear as to why BRAF inhibitors were infrequently used in this population, one explanation may be the underwhelming efficacy of BRAF inhibition in management of GI cancers when compared to melanoma. In trials assessing the efficacy of BRAF inhibition in combination with MEK inhibition in the treatment of melanoma, response rates of 63-69% were seen.^36-39^ However, in the case of CRC, the BEACON trial found that those who received a combination of encorafenib plus cetuximab (EGFR inhibitor) exhibited an objective response rate of 20%.^3^ In the case of biliary tract cancers, dabrafenib plus trametinib has resulted in an ORR of 51%, however, these were all partial responses in the ROAR trial.^15^ Prospective data for the use of BRAF inhibitors in other GI malignancies is limited. Additionally, BRAF inhibitors have only been approved in patients with GI malignancies at the time of disease progression, which may also reduce the number of patients receiving therapy. An additional consideration is the relatively recent approval of BRAF inhibitors in non-CRC GI malignancies which may impact rates of utilization.

Limitations of this study include its retrospective nature and lack of clinical data for all patients, particularly those with HCC. Additionally, the relative infrequency of various *BRAF* mutations in these malignancies does make it more challenging to draw definitive conclusions comparing rwOS, particularly given the high censorship rate in the cohorts presented here. In this study, we were not able to elucidate the utility of *BRAF* inhibitors in this patient population as too few in our study cohort were treated with *BRAF* inhibitors. Further, while we report prognostic implications of various *BRAF* mutations among non-CRC GI malignancies, implications of these mutations may change with increased utilization of BRAF inhibitors among patients with *BRA*F^V600E^ mutations. Additionally, missingness of data may occur in real-world datasets due to lack of continuous enrollment in health plans and/or loss-to-follow-up; rwOS should be interpreted accordingly.

## Conclusions

In this study, we demonstrate the feasibility of the detection of classified *BRAF* alterations among patients with non-CRC GI malignancies via liquid biopsy. To the best of our knowledge, this is the largest study to assess the prevalence and potential impact of *BRAF* alterations in patients with non-CRC GI cancers, and one of the only studies to publish prevalence for hepatocellular carcinoma. While these alterations are relatively rare, they may have prognostic implications, as well as implications in treatment considering recent FDA tumor-agnostic approval of dabrafenib and trametinib. This study further demonstrates the importance of biomarker testing and the utility of liquid biopsy in patients with advanced GI cancers and the ongoing need to develop and assess the impact of *BRAF*-targeted therapies on patient outcomes in these subpopulations.

## Supplementary Material

oyaf044_suppl_Supplementary_Tables_S1

oyaf044_suppl_Supplementary_Figures_S1

## Data Availability

The data underlying this article will be shared on reasonable request to the corresponding author.

## Notes

**Prior Presentation:** Presented in part at the 2023 ASCO Gastrointestinal Cancers Symposium.
