# Supplementary material for: Frequency and outcomes of BRAF alterations identified by liquid biopsy in metastatic, non-colorectal gastrointestinal cancers
Source: Oncologist. 2025 Mar 31;30(3):oyaf044. doi: 10.1093/oncolo/oyaf044 (PMC11957259; doi:10.1093/oncolo/oyaf044)
Supplement: oyaf044_suppl_Supplementary_Tables_S1 [file oyaf044_suppl_supplementary_tables_s1.docx]

**Table S1.** BRAF alterations by Class

| **Class** | **BRAF Alteration** |
| --- | --- |
| Class I^1^ | V600E  V600K/R/L/D |
| Class II^1^ | Q257R  I463S  G464V/R  G469A/V/R  V471F  L485F  L485_P490delinsY  N486_P490del  V487_P492delinsA  K499E  L505H/F  E586K  L597R/S/W  T599dup  T599R  V600_K601delinsE  V600_K601delinsEN  V600_S605delinsEISRWR  K601E/N/Q/T |
| Class III^1^ | D287H  D594N/V  G466V/E/R/A  S467L  G469E  K483E  N581S/I/Y/K  D594A/E/H  F595L  G596R/C |
| Other Predicted Driver | S122P  T241M  L245F/V  F247C/I/L/V  A246P Q257L  L285_P490delinsF  D287A/G  P367L  E451K V459A  R462T  S467L  F468C/S  G469_T470delinsA  G469R/S/T  V471F  Y472C  K483Q/R L485M/S/W  N486_A489delinsK  N486_P490del  N486_Q493delinsTPE  N486_Q493delinsTPK  A489_Q493del  T488_Q493delinsE  K499N/R  R506_K507insVLR  H574L  N581T/Y  E586Q  D594A/G  F595_V600delinsL  G596S/V  L597V/Q  A598dup  A598G T599del  T599I  V600_R603delinsESG  V600_Q604delinsR  R671Q  R682W  E695D/K  H725R  P731S |

Class I-III were used for outcomes analysis
Other predicted drivers were included in prevalence analysis only
