# Supplementary material for: Frequency and outcomes of BRAF alterations identified by liquid biopsy in metastatic, non-colorectal gastrointestinal cancers
Source: Oncologist. 2025 Mar 31;30(3):oyaf044. doi: 10.1093/oncolo/oyaf044 (PMC11957259; doi:10.1093/oncolo/oyaf044)

**Figure S1.** Co-occurring alterations with BRAF V600E in the Outcomes Cohort

The oncoprpints below (A, B, C) are designed to show co-occurring alterations for each patient and across the disease-specific cohort (cholangiocarcinoma, pancreatic cancer and gastroesophageal cancer respectively).

- Each column is a patient’s genomic result.
- The left axis with percent values represents the frequency a gene is altered in the cohort (100% of patients had BRAF alterations and 41% of patients had TP53 alterations in Figure A).
- The heatmap at the right of the listed genes displays the impacted number of patients with a given gene altered (e.g., 155 had BRAF alterations, ~50 had TP53 alterations).
- The heatmap on top of the oncoprint represents a per-patient alteration count that corresponds to the same column of a patient’s genomic findings (e.g. the first patient in the first column had 5 alterations, the second in the second column had 7). Alterations with a frequency <1% in the cohort may not be shown.
- The colors of red, green, orange and blue correspond to alteration type, as listed in the key on each oncoprint.

A.


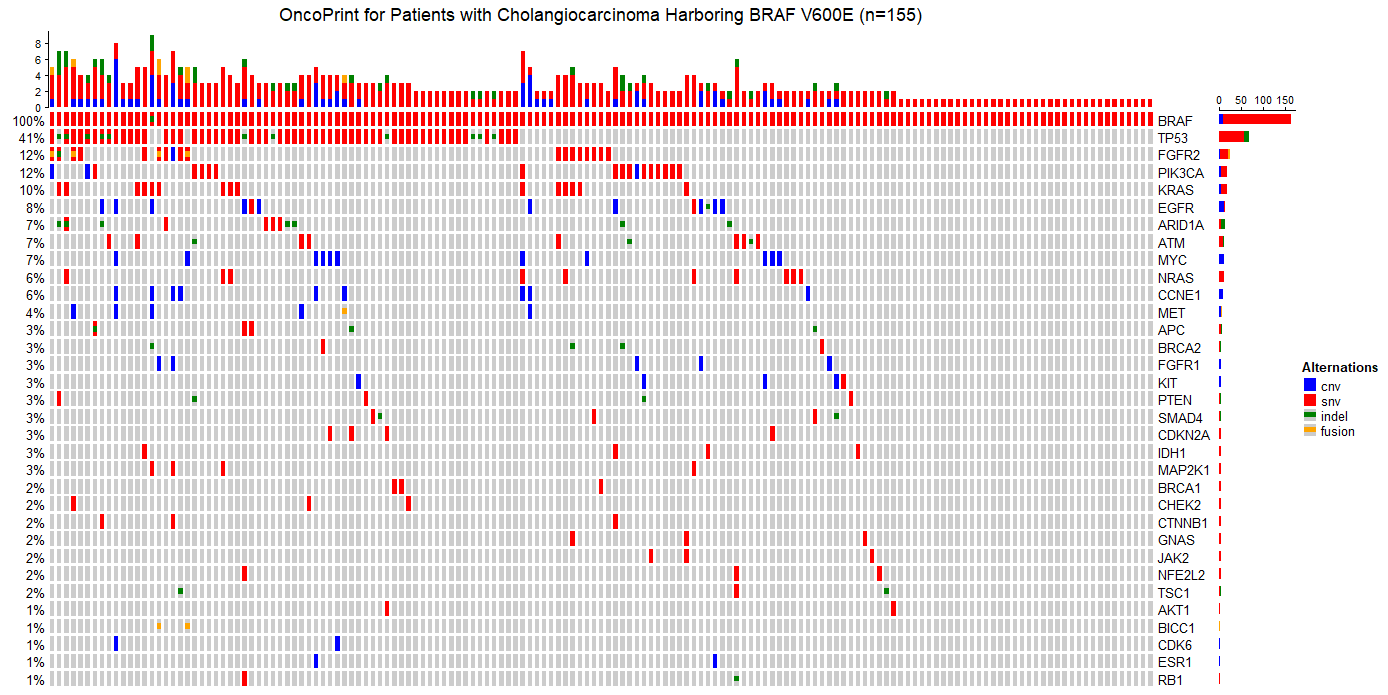


B.


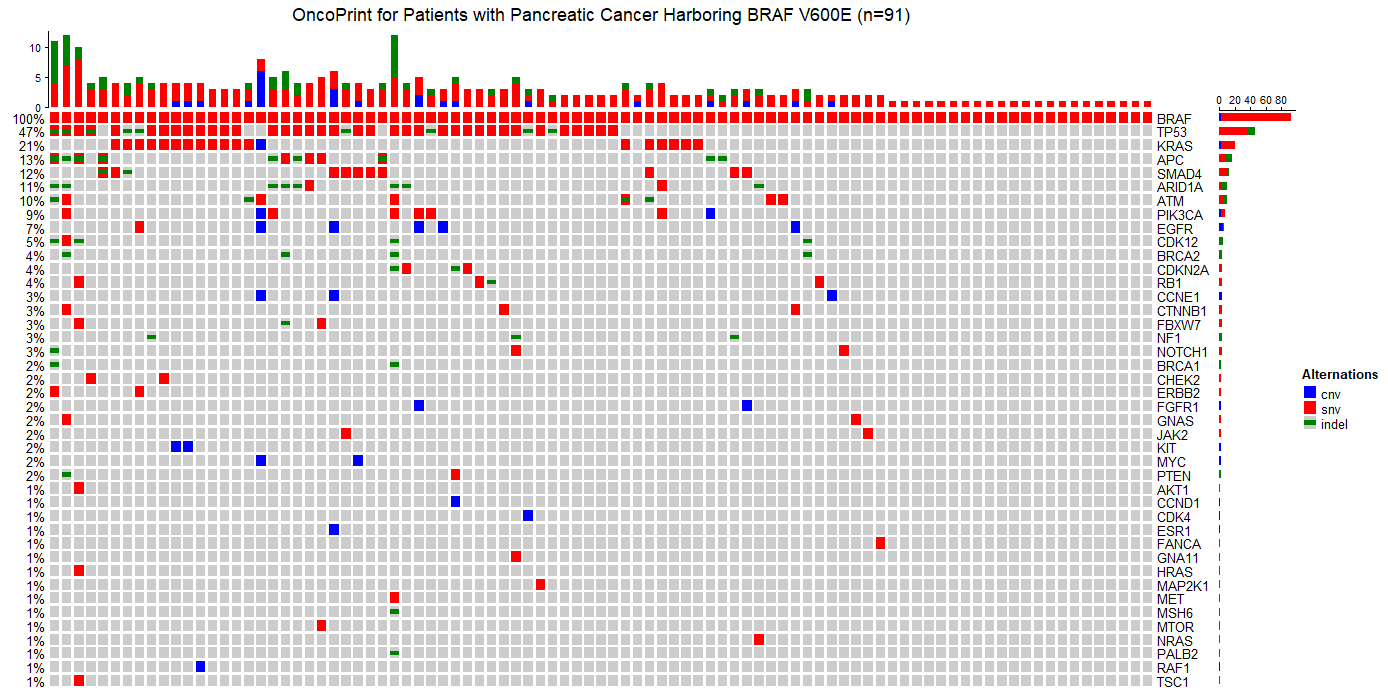


C.

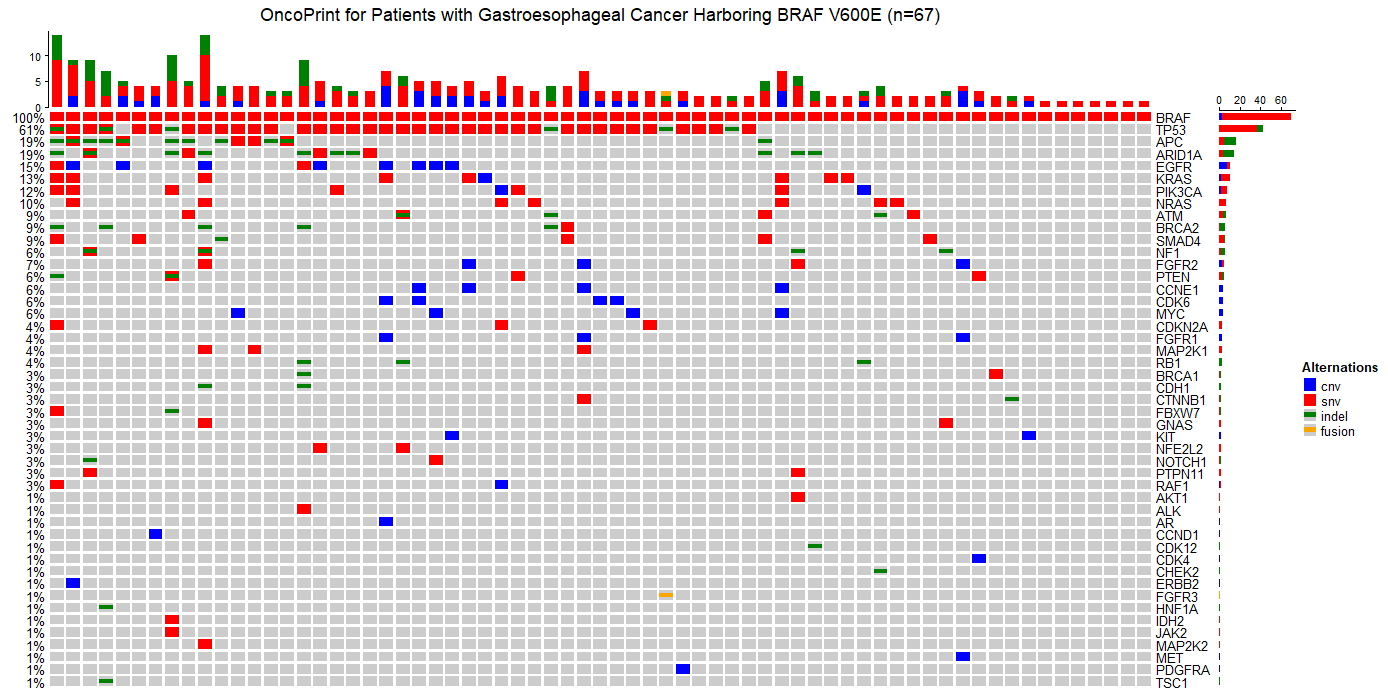

Supplement: oyaf044_suppl_Supplementary_Figures_S1 [file oyaf044_suppl_supplementary_figures_s1.docx]
